# Inconsistent classification of unexplained sudden deaths in infants and children hinders surveillance, prevention and research: recommendations from The 3rd International Congress on Sudden Infant and Child Death

**DOI:** 10.1007/s12024-019-00156-9

**Published:** 2019-09-09

**Authors:** Richard D. Goldstein, Peter S. Blair, Mary Ann Sens, Carrie K. Shapiro-Mendoza, Henry F. Krous, Torleiv O. Rognum, Rachel Y. Moon, The 3rd International Congress on Sudden Infant and Child Death, Robert N. Anderson, Peter S. Blair, Elizabeth A. Bundock, Laura G. Crandall, Robert A. Darnall, Richard D. Goldstein, Fern Hauck, Robin L. Haynes, Barbara Himes, Susan Hollander, Ingrid A. Holm, Christine Keywan, Henry F. Krous, Betty McEntire, Edwin A. Mitchell, Rachel Y. Moon, Torleiv O. Rognum, Mary Ann Sens, Carrie K. Shapiro-Mendoza, Jan Sperhake, Barbara A. Sampson, Mark Super

**Affiliations:** 1 Boston Children’s Hospital and Harvard Medical School, 300 Longwood Avenue, BCH3201, Boston, MA 02138 USA; 2 Bristol Medical School, University of Bristol, Bristol, UK; 3 University of North Dakota School of Medicine and Health Sciences, Grand Forks, ND USA; 4 Centers for Disease Control and Prevention, Atlanta, GA USA; 5 Rady Children’s Hospital and UCSD School of Medicine, San Diego, CA USA; 6 Oslo University Hospital and University of Oslo, Oslo, Norway; 7 University of Virginia School of Medicine, Charlottesville, VA USA

**Keywords:** Sudden infant death syndrome, Sudden unexplained death in childhood, Sudden unexplained infant death, Sudden unexpected infant death, Postneonatal mortality, Accidental suffocation and strangulation in bed, Undetermined infant death

## Abstract

This report details the proceedings and conclusions from the 3rd International Congress on Unexplained Deaths in Infants and Children, held November 26–27, 2018 at the Radcliffe Institute at Harvard University. The Congress was motivated by the increasing rejection of the diagnosis Sudden Infant Death Syndrome (SIDS) in the medical examiner community, leading to falsely depressed reported SIDS rates and undermining the validity and reliability of the diagnosis, which remains a leading cause of infant and child mortality. We describe the diagnostic shift away from SIDS and the practical issues contributing to it. The Congress was attended by major figures and opinion leaders in this area from countries significantly engaged in this problem. Four categories (International Classification of Diseases (ICD)-11 categories of MH11, MH12, MH14, PB00-PB0Z) were recommended for classification, and explicit definitions and guidance were provided for death certifiers. SIDS was reframed as unexplained sudden death in infancy or SIDS/MH11 to emphasize that either term signifies the lack of explanation following a rigorous investigation. A distinct category for children over the age of 1 was recommended (MH12). Definitions and exclusions were provided for the alternative categories of accidental asphyxia and undetermined. As recommended, unexplained sudden death in infancy or SIDS on a death certificate will code a unique, trackable entity, accurately reflecting the inability to determine a definitive explanation, while satisfying surveillance needs and reliable identification for research efforts. The conclusions will be submitted to the World Health Organization for inclusion in the upcoming ICD-11.

## The problem

Many would consider sudden infant death syndrome (SIDS) to be an uncontroversial, largely solved problem of infant safety, yet it remains a leading cause of infant mortality in the developed world. Efforts in diagnosis, surveillance, research, and prevention are complicated by substantial divergence in certification and coding of these deaths [1, 2]. At the center of this divergence is the very concept of SIDS. Although the term SIDS is endorsed by the World Health Organization (WHO) and is broadly used by physicians, researchers, and the public, it is increasingly avoided by forensic pathologists in many jurisdictions who determine cause and certify sudden deaths in infants [1].

**Fig. 1:**
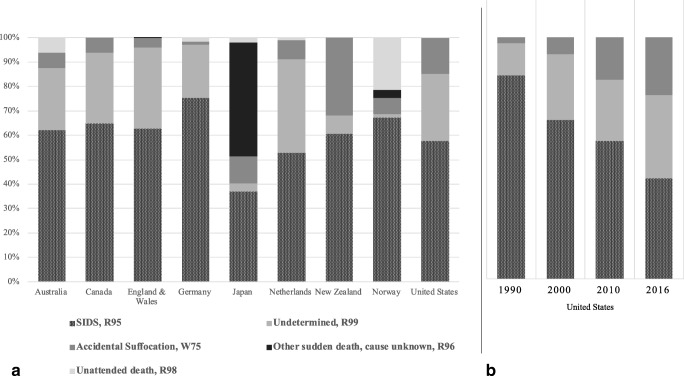
Evidence for diagnostic shift. Multinational comparison of diagnostic preferences (a) and time study of US trends (b). **a** International classification of diseases-10 (ICD-10) codes as percentages of the total sudden unexpected deaths in infancy per country, 2002–2010. Modified from Taylor et al. [4]. **b** Proportionate use of unexplained infant death categories in the United States, 1990–2016. Modified from Erck Lambert et al. [31]

There are three basic issues that have contributed to difficulties with the diagnosis of SIDS. First, there have long been questions among forensic pathologists about the evidence required to apply a diagnosis of exclusion. Even with formal death scene investigations, evidence often has uncertain implications. Second, it has become the practice in many jurisdictions for forensic pathologists to refrain from using the diagnosis of SIDS under any circumstances. Third, once deaths have been certified by pathologists, surveillance requires an interface between death certificates and ICD codes and coding algorithms, a process that sometimes leads to an entered diagnostic code (classification) that is different than the certifier’s intention. These factors contribute to diagnostic shift, where reported declines in SIDS rates are exaggerated by the attribution of these deaths to other causes [3]. This reassignment, towards “undetermined/unascertained” or “asphyxia” occurring in a sleep environment, serves to underestimate the actual mortality of what was once considered SIDS. The phenomenon is seen in virtually every advanced economy [4], although lessened in Scandinavia due to regional consensus [5] (Fig. 1a). While some countries report decreases in SIDS since initial declines in the early 1990’s, one implication of this reassignment, particularly in the United States (US), is that over half of what was once SIDS is now counted otherwise (Fig. 1b). Alternative classifications, each with different terminology and definitions have not achieved consensus, but instead have contributed to discordant use of terminology and acronyms (Table 1). For example, although the acronyms SUID or SUDI are often assumed to be interchangeable, the “U” modifying sudden infant death may represent unexpected, undetermined, unknown, unexplained, or unascertained in actual usage [6–8]. While SUID/SUDI has its origins as an umbrella term for the initial presentation of ultimately explained or unexplained infant deaths, it is commonly used by death certifiers as an alternative to a final SIDS diagnosis. Interested parties have failed to agree on the definition and terminology for the leading cause of postneonatal mortality [6, 7, 9–14]. Current classification practices have poor reliability and undermine accounting of progress [15].

**Table 1:** Terminology and previously proposed diagnostic schema

	Sudden infant death syndrome (SIDS)	Sudden unexpected infant death (SUID)	Sudden unexplained infant death (SUID)	Sudden unexpected death in infancy (SUDI)
Beckwith, 2nd International SIDS Conference^6^	X			
Willinger, US National Institute for Child Health and Development^7^	X			
Gilbert^8^		X		
Krous^9^	X			
Corey, National Association of Medical Examiners^10^			X	
Randall^13^		X		
Fleming and Blair, Avon^12^				X
Shapiro-Mendoza, Centers for Disease Control SUID Registry^11^		X		

The international adoption of SIDS terminology in 1969 was intended to identify infants dying from unknown causes in order to investigate their deaths as a distinct but unexplained entity in infancy. The core phenomenon included the relative sparing of deaths in the newborn period, concentration of unexplained mortality between 2 and 6 months, a relationship with sleep, and non-specific but prevalent findings on autopsy. Ensuing epidemiological research found risks associated with infant sleep environments [16], which in turn spurred public health efforts and led to a worldwide transformation in infant care practices and the promotion of formal death scene investigations [17]. Indications of underlying pathology and biological processes were discovered in many of these infants, including intrathoracic petechiae [18], brainstem gliosis [19], brainstem serotonin deficiencies [20], epilepsy-associated neuropathological findings [21], associations with prenatal alpha-fetoprotein [22], associations with antenatal alcohol and tobacco exposure [23], and genetic findings [24, 25]. However, diverging opinions emerged in interpreting the relative roles of extrinsic factors and underlying biological processes.

The impasse was not without reason. The importance of autopsy findings and sleep environment observations are often difficult to estimate, and terminal events generally remain a matter of speculation. The triple risk theory [26], the leading etiologic framework for SIDS, proposes that extrinsic factors become lethal in an infant with intrinsic vulnerabilities when challenged at critical developmental ages, but those extrinsic factors are not uniformly lethal. Multiple environmental risk factors for SIDS (prone sleep position, bed/surface sharing, soft bedding or blankets) are often found, but interpreting whether the evidence confirms accidental asphyxia is challenging and inconsistently concluded. Furthermore, some autopsy findings are non-specific and may be identical in deaths from SIDS, asphyxia (accidental or homicidal), or natural diseases such as arrhythmias and metabolic disorders. Additionally, while extrinsic factors in an infant’s sleep environment are typically noted, features that may influence underlying biologic processes often do not receive attention (smoking during pregnancy, in utero alpha-fetoprotein levels, brainstem serotonin levels, epilepsy in-situ). If there is agreement that there exists an incompletely understood entity involving intrinsic biological and environmental factors, there is disagreement about how to assess these factors uniformly.

There are difficulties related to classification. Coding algorithms may assign these deaths to classifications that the certifier did not intend. For example, a death certificate reading undetermined/unascertained with mention of possible asphyxia due to bed sharing is coded as accidental suffocation and strangulation in bed (ASSB) by WHO coding algorithms, in effect elevating risk factor to cause in classification. A case with identical details might be certified as SIDS or undetermined without mention of risk factors or possible cause of death by other certifiers. Finally, there are important human aspects of this problem, devastating to a family on many levels. On the level of those affected, bereaved parents associate blame and judgment with the use of different terms [27], with ASSB and undetermined cause of death carrying a stigma of implied blame, even when evidence does not support that parents have caused harm. Parents suffer from severe grief-related symptoms [28], and few options are available to obtain enhanced diagnostics for better explanations, despite recent advances in undiagnosed diseases [29].

## The proceedings

The World Health Organization periodically releases an updated manual for the international classification of diseases (ICD). The current revision (ICD-11) is in its final stages before planned implementation in 2022. The 3rd International Congress on Sudden Infant and Child Death was convened to align the current scientific understanding of sudden unexpected death in infancy/childhood with certification and coding nomenclature. It sought a practical consensus on essential characteristics of this category of mortality and the best nomenclature to reflect this. In attendance were representatives of the Centers for Disease Control and Prevention (US), National Association of Medical Examiners (US, Canada), American Academy of Pediatrics (US), the International Society for the Study and Prevention of Perinatal and Infant Death (International), leading basic science and epidemiological researchers from around the world (US, UK, Norway, Germany, New Zealand), and included pediatricians, pediatric pathologists, forensic pathologists, family physicians, epidemiologists, statisticians, researchers, and parent representatives.

**Fig. 2:**
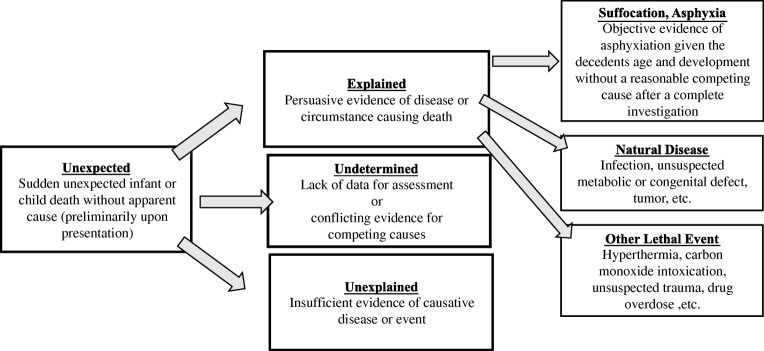
Consensus for recommended use of the terms unexpected, unexplained, undetermined, and explained subsets. The Congress reviewed, clarified and recommended terminology relative to the process of case determination

It was first necessary to agree on the precise use of the terms unexpected, unexplained, unascertained and undetermined. As originally conceived, SIDS refers to “the sudden death of any infant or young child, which is unexpected by history, and in which a thorough post-mortem investigation fails to demonstrate an adequate cause for death” [9]. In recent years, a thorough post-mortem investigation has included appropriate laboratory testing [7]. By convention, “unexpected” denotes a preliminary designation pending investigation. If the investigation does not reveal a cause, cases are designated as “unexplained”. Deaths where investigations are incomplete or competing defined causes preclude final certification warrant the designation “undetermined” (Fig. 2).

Differing perspectives on the evidence required before using the diagnostic categories of undetermined or suffocation/asphyxia death were discussed. Despite diagnostic criteria that include complete investigations, actual practice and available resources vary. Death certification involves a determination of both cause and manner, and the Radcliffe Congress placed emphasis on objective evidence to support conclusions for cause. The presence of sleep environment risk factors without adequate evidence for airway obstruction or chest wall compression were considered insufficient to certify a death due to asphyxia. The need for stringent criteria before using “undetermined” or asphyxia, categories that shift away from SIDS, was emphasized, along with the need for precise, descriptive terminology. There was affirmation that once abuse, neglect and trauma are eliminated, the needs of the family should be prioritized by clearly explaining the strength of the evidence for why the infant died and potential implications to them.

## Recommendations

After two days of debate, the following conclusions were unanimously reached (Table 2, with definitions for death certifiers in the caption). Refinements were proposed to four relevant diagnostic categories (ICD-11 categories of MH11, MH12, MH14, PB00-PB0Z) to be recommended for content enhancement to the WHO, and explicit definitions and guidance were provided for death certifiers. A title change from “SIDS” to “Unexplained Sudden Death in Infancy or SIDS”, using the definition established by the (US) National Association of Medical Examiners [7], was recommended for the MH11 category to emphasize that either term signifies the lack of explanation following a rigorous investigation. This definition describes the death of an infant less than 1 year of age, in which investigation, autopsy, medical history review, and appropriate laboratory testing fails to identify a specific cause of death, and includes cases that meet the definition of sudden infant death syndrome. The use of “unexplained sudden death in infancy” without acronym acknowledged the difficulties of having acronyms with different interpretations and also addressed objections by some medical examiners that the term” SIDS” should not be used under any circumstances. The simultaneous inclusion of “SIDS” within the MH11 category confirmed both the historical importance of this term and the recognition that there may be identifiable intrinsic vulnerabilities contributing to the death whose examination is not included in a standard investigation (e.g. serotonin deficiencies in the ventral medulla, epilepsy-in-situ in the dentate gyrus, pathogenic genetic variants) or yet-to-be identified vulnerabilities that are the focus of future research. A subcategory MH11.1 for unexplained sudden deaths in infancy or SIDS without mention of autopsy recognizes that there are communities and cultures within which autopsies are not regularly completed due to lack of resources or cultural norms (e.g. religious objections); inclusion of this category will allow these deaths to be included for research and statistical purposes. A formalized emphasis for similar unexplained sudden deaths in children over the age of 1, beyond “other sudden death,” within the MH12 category that includes adults, was also recommended, to improve surveillance of an increasingly recognized category of unexplained child deaths. Explicit guidance was provided for the use of the alternative categories of “accidental asphyxia” and “undetermined”, with recommendations for essential requirements before their use in these cases. Acronyms were discouraged to prevent future misconceptions. Unexplained sudden death in infancy or SIDS on a death certificate will code a unique, trackable entity, accurately reflecting the inability to determine a definitive explanation, while satisfying surveillance needs and reliable identification for research efforts. These recommendations will be immediately proposed to the WHO for the ICD-11. In addition, the recommendations will be disseminated among international forensic pathology and pediatric organizations.

**Table 2:** Consensus classification of unexplained sudden deaths in infants and children

Proposed ICD-11 Code	Current ICD-10 Code	Proposed ICD-11 Stem Code/Classification	ICD-10 Classification	Notes for Death Certifiers
MH11	R95	Unexplained sudden death in infancy or Sudden Infant Death Syndrome	Sudden Infant Death Syndrome	a
MH11.0	R95.0	Unexplained sudden death in infancy or Sudden Infant Death Syndrome, with mention of autopsy	Sudden Infant Death Syndrome	a
MH11.1	R95.9	Unexplained Sudden Death in Infancy or Sudden Infant Death Syndrome, without mention of autopsy	Sudden Infant Death Syndrome	a
MH12	R96	Unexplained sudden death in children and adults	Other sudden death, cause unknown	b
MH12.0		Unexplained sudden death in children and adults, with mention of autopsy		b
MH12.1		Unexplained sudden death in children and adults, without mention of autopsy		b
MH14	R99	Other Ill-Defined or Unspecified Causes of Death (Undetermined)	Other Ill-Defined or Unspecified Causes of Death	c, d
PB00-PB0Z	W75-W84	Unintentional threat to breathing (accidental asphyxia)	Unintentional threat to breathing by external compression of airways or chest; Unintentional threat to breathing by unspecified means	e, f

a:The sudden unexpected death of an apparently healthy infant under one year of age that remains unexplained after a thorough case investigation, including performance of a complete autopsy with ancillary testing, examination of the death scene, and review of the clinical history

b: The sudden unexpected death of a person one year of age or older that remains unexplained after a thorough case investigation, including performance of a complete autopsy with ancillary testing, and review of the clinical history and circumstances of death

c: Cases may be certified as undetermined when: the investigation, death scene examination, or autopsy was substantially limited, incomplete or insufficient, for example legal/religious restrictions, delayed report of death that limits scene investigation, or decomposition; or when inconsistent accounts or other findings raise competing conclusions about the cause of death

d: Infant deaths with adequate death scene investigation and autopsy, with a history of bed/sleep surface sharing, soft bedding, or non-supine sleep, and without physical evidence of asphyxia, may be more appropriately certified as unexplained sudden death in infancy or sudden infant death syndrome

e: Certification of asphyxia: Adequate evidence must be documented to substantiate asphyxiation, given the decedent’s age and stage of development. There cannot be a reasonable competing cause of death after a complete autopsy with ancillary testing, examination of the death scene (with a doll re-enactment when appropriate), and review of the clinical history

f: In infants, bed/sleep surface sharing, soft bedding, or prone sleep, without adequate evidence for airway obstruction or chest wall compression, are insufficient to certify a death as due to asphyxia. These deaths may be more appropriately certified as unexplained sudden death or SIDS. The use of “possible” or “probable” asphyxia will result in the death being classified as asphyxia

## Conclusions

Sudden death in infants remains a distinct and tragic category of death, and terminal events remain a matter of speculation in most cases. While different medical constituencies may prioritize different aspects of unexplained deaths, progress was made to increase the reliability of the category. With broad representation and an insistence on consensus, the Radcliffe Congress succeeded in 1) articulating definitions for the classification of unexplained sudden deaths in infants and children; 2) discriminating categories with explicit guidance for death certifiers in the use of alternative diagnoses; and 3) providing an approach for dissemination. If implemented, the reported incidence of unexplained sudden death in infancy or sudden infant death syndrome/MH11 will more accurately account for cases that would otherwise be lost in diagnostic shift. The diagnostic category of “unexplained sudden death in children and adults/MH12” will improve international surveillance of similar deaths in children beyond infancy [30]. The mutual understanding gained at the Radcliffe Congress promises better future collaboration in addressing this persistent, heartbreaking matter of infant and child mortality.

## References

[1] Shapiro-Mendoza CK, Parks SE, Brustrom J, Andrew T, Camperlengo L, Fudenberg J, et al. Variations in cause-of-death determination for sudden unexpected infant deaths. Pediatrics. 2017;140. 10.1542/peds.2017-0087. 10.1542/peds.2017-0087 PMC5599098 28759406

[2] Gould SJ, Weber MA, Sebire NJ (2010). Variation and uncertainties in the classification of sudden unexpected infant deaths among paediatric pathologists in the UK: findings of a National Delphi Study. J Clin Pathol.

[3] Shapiro-Mendoza CK, Tomashek KM, Anderson RN, Wingo J (2006). Recent national trends in sudden, unexpected infant deaths: more evidence supporting a change in classification or reporting. Am J Epidemiol.

[4] Taylor BJ, Garstang J, Engelberts A, Obonai T, Cote A, Freemantle J (2015). International comparison of sudden unexpected death in infancy rates using a newly proposed set of cause-of-death codes. Arch Dis Child.

[5] Vege A, Rognum TO (1997). Use of new Nordic criteria for classification of SIDS to re-evaluate diagnoses of sudden unexpected infant death in the Nordic countries. Acta Paediatr.

[6] Shapiro-Mendoza CK, Camperlengo L, Ludvigsen R, Cottengim C, Anderson RN, Andrew T (2014). Classification system for the sudden unexpected infant death case registry and its application. Pediatrics..

[7] Corey T, Hanzlick R, Howard J, Nelson C, Krous H (2007). A functional approach to sudden unexplained infant deaths. Am J Forensic Med Pathol.

[8] Mitchell EA, Thompson JM, Zuccollo J, MacFarlane M, Taylor B, Elder D (2017). The combination of bed sharing and maternal smoking leads to a greatly increased risk of sudden unexpected death in infancy: the New Zealand SUDI nationwide case control study. NZ Med J.

[9] Bergman AB, Beckwith JB, Ray CG (1970). Sudden infant death syndrome: proceedings of the second international conference on causes of sudden death in infants.

[10] Willinger M, James LS, Catz C (1991). Defining the sudden infant death syndrome (SIDS): deliberations of an expert panel convened by the National Institute of Child Health and Human Development. Pediat Pathol.

[11] Gilbert R, Rudd P, Berry PJ, Fleming PJ, Hall E, White DG (1992). Combined effect of infection and heavy wrapping on the risk of sudden unexpected infant death. Arch Dis Child.

[12] Leach CE, Blair PS, Fleming PJ, Smith IJ, Platt MW, Berry PJ, et al. Epidemiology of SIDS and explained sudden infant deaths. CESDI SUDI Research Group. Pediatrics. 1999;104:e43. 10.1542/peds.104.4.e43 10506268

[13] Krous HF, Beckwith JB, Byard RW, Bajanowski T, Corey T, Rognum TO (2004). Sudden infant death syndrome and unclassified sudden infant deaths: a definitional and diagnostic approach. Pediatrics..

[14] Randall BB, Wadee SA, Sens MA, Kinney HC, Folkerth RD, Odendaal HJ (2009). A practical classification schema incorporating consideration of possible asphyxia in cases of sudden unexpected infant death. Forensic Sci Med Pathol.

[15] Goldstein RD, Trachtenberg FL, Sens MA, Harty BJ, Kinney HC. Overall postneonatal mortality and rates of SIDS. Pediatrics. 2016;137. 10.1542/peds.2015-2298. 10.1542/peds.2015-2298 26634772

[16] Mitchell EA, Tuohy PG, Brunt JM, Thompson JM, Clements MS, Stewart AW (1997). Risk factors for sudden infant death syndrome following the prevention campaign in New Zealand: a prospective study. Pediatrics..

[17] Moon RY. Task force on sudden infant deaths. SIDS and other sleep-related infant deaths: evidence base for 2016 updated recommendations for a safe infant sleeping environment. Pediatrics. 2016;138. 10.1542/peds.2016-2940. 10.1542/peds.2016-2940 27940805

[18] Krous HF, Haas EA, Chadwick AE, Masoumi H, Stanley C (2008). Intrathoracic petechiae in SIDS: a retrospective population-based 15-year study. Forensic Sci Med Pathol..

[19] Naeye RL (1976). Brain-stem and adrenal abnormalities in the sudden-infant-death syndrome. Am J Clin Pathol.

[20] Duncan JR, Paterson DS, Hoffman JM, Mokler DJ, Borenstein NS, Belliveau RA (2010). Brainstem serotonergic deficiency in sudden infant death syndrome. JAMA..

[21] Kinney HC, Cryan JB, Haynes RL, Paterson DS, Haas EA, Mena OJ (2015). Dentate gyrus abnormalities in sudden unexplained death in infants: morphological marker of underlying brain vulnerability. Acta Neuropathol.

[22] Smith GC, Wood AM, Pell JP, White IR, Crossley JA, Dobbie R (2004). Second-trimester maternal serum levels of alpha-fetoprotein and the subsequent risk of sudden infant death syndrome. New Eng J Med.

[23] Kinney HC, Randall LL, Sleeper LA, Willinger M, Belliveau RA, Zec N (2003). Serotonergic brainstem abnormalities in Northern Plains Indians with the sudden infant death syndrome. J Neuropathol Exp Neurol.

[24] Brownstein C, Goldstein RD, Thompson C, Haynes RL, Giles E, Sheidley B (2018). SCN1A variants associated with sudden infant death syndrome. Epilepsia..

[25] Mannikko R, Wong L, Tester DJ, Thor MG, Sud R, Kullmann DM (2018). Dysfunction of NaV1.4, a skeletal muscle voltage-gated sodium channel, in sudden infant death syndrome: a case-control study. Lancet..

[26] Filiano JJ, Kinney HC (1994). A perspective on neuropathologic findings in victims of the sudden infant death syndrome: the triple-risk model. Biol Neonate.

[27] Crandall LG, Reno L, Himes B, Robinson D (2017). The diagnostic shift of SIDS to undetermined: are there unintended consequences?. Acad Forens Path.

[28] Goldstein RD, Lederman RI, Lichtenthal WG, Morris SE, Human M, Elliott AJ, et al. The grief of mothers after the sudden unexpected death of their infants. Pediatrics. 2018;141. 10.1542/peds.2017-3651. 10.1542/peds.2017-3651 PMC6173829 29712764

[29] Goldstein RD, Nields HM, Kinney HC. A new approach to the investigation of sudden unexpected death. Pediatrics. 2017;140. 10.1542/peds.2017-0024. 10.1542/peds.2017-0024 28679642

[30] Krous HF, Chadwick AE, Crandall L, Nadeau-Manning JM (2005). Sudden unexpected death in childhood: a report of 50 cases. Pediat Dev Pathol.

[31] Erck Lambert AB, Parks SE, Shapiro-Mendoza CK. National and state trends in sudden unexpected infant death: 1990-2015. Pediatrics. 2018;141. 10.1542/peds.2017-3519. 10.1542/peds.2017-3519 PMC6637428 29440504

